# Speculative computing for AAFM solutions in large-scale product configurations

**DOI:** 10.1038/s41598-024-61647-6

**Published:** 2024-05-16

**Authors:** Cristian Vidal-Silva, Vannessa Duarte, Jesennia Cárdenas-Cobo, Iván Veas

**Affiliations:** 1 School of Videogame Development and Virtual Reality Engineering, Faculty of Engineering, University of Talca, Av. Lircay S/N, 3460000 Maule, Talca Chile; 2 Escuela de Ciencias Empresariales, Universidad Católica del Norte, Larrondo 1280, 178142 Coquimbo, Coquimbo Chile; 3 Facultad de Ciencias e Ingenierías, Universidad Estatal de Milagro, Cdla. Universitaria Km1/2 vía Km 26, 091706 Milagro, Guayas Ecuador; 4 Departamento de Administración, Facultad de Economía y Administración, Universidad Católica del Norte, Av. Angamos 0610, 1270709 Antofagasta, Antofagasta Chile

**Keywords:** Speculative programming, AAFM, Conflict detection, Diagnosis detection, Configuration, Computational science, Software, Applied mathematics

## Abstract

Parallel computing is a current algorithmic approach to looking for efficient solutions; that is, to define a set of processes in charge of performing at the same time the same task. Advances in hardware permit the massification of accessibility to and applications of parallel computing. Nonetheless, some algorithms include steps that require or depend on the results of other steps that cannot be parallelized. Speculative computing allows parallelizing those tasks and reviewing different execution flows, which can involve executing invalid steps. Speculative computing solutions should reduce those invalid flows. Product configuration refers to selecting features from a set of available options respecting some configuration constraints; a not complex task for small configurations and models, but a complex one for large-scale scenarios. This article exemplifies a videogame product line feature model and a few configurations, valid and non-valid, respectively. Configuring products of large-scale feature models is a complex and time-demanding task requiring algorithmic solutions. Hence, parallel solutions are highly desired to assist the feature model product configuration tasks. Existing solutions follow a sequential computing approach and include steps that depend on others that cannot be parallelized at all, where the speculative computing approach is necessary. This article describes traditional sequential solutions for conflict detection and diagnosis, two relevant tasks in the automated analysis of feature models, and how to define their speculative parallel version, highlighting their computing improvements. Given the current parallel computing world, we remark on the advantages and current applicability of speculative computing for producing faster algorithmic solutions.

## Introduction

Parallel computing leverages the power of multiple processors or computing elements to execute tasks simultaneously, thereby enhancing the performance and speed of complex computations^1^. This technology has gained significant importance in recent years, as it addresses the ever-increasing demand for processing power and data analysis in various fields, from scientific research^2,3^, artificial intelligence^4^, financial modeling^5^ and video rendering^6^. Parallel computing offers several notable advantages but presents particular challenges that must be carefully considered.

Regarding parallel computing benefits, improvements in processing speed, computing performance, scalability, adaptability, resource utilization, problem-solving, and redundancy with fault tolerance are highlighted. Chandrashekhar and Sanjay^7^ emphasize that parallel computing can dramatically increase processing speed and performance, particularly relevant for real-time applications and simulations. Robey and Zamora^8^ note that parallel computing easily scales by adding more processors or nodes, allowing adaptation to changing demands and avoiding bottlenecks. Parallel computing optimizes hardware resource utilization, minimizing idle time and ensuring available computing power is harnessed^9^. Wu et al.^10^ mention that parallel computing is ideal for solving complex problems by distributing smaller, independent subproblems across multiple processors. Huang, Coolen, and Coolen-Maturi^11^ point out that parallel systems can achieve fault tolerance through redundancy, redistributing workload if a processor fails. However, parallel computing presents challenges such as programming complexity, software and hardware costs, load balancing, data dependency, and Amdahl’s Law^12^. A common strategy for enhancing scalability is task-based algorithms, dissociating algorithmic parallelization from code, data structure, and computational cores^13,14^.

Speculative programming, an innovative computing approach, anticipates and addresses potential issues preemptively in software systems^15^. Speculative execution, as described by^16^, aims to speed up program execution by running code segments before their utility is known. Then, speculative execution is the pre-execution or pre-calculation of results that can contribute to achieving the expected computation outcome^17^. Thread Level Speculation (TLS), represented by speculative executions^18^, tackles the limitations of static approaches through parallel task execution^19^. However, speculative executions can yield effective or non-valid outcomes, potentially impacting program execution time^19^.

Variability-intensive systems (VIS) focus on variability management and product configuration^15,20,21^. VIS product configuration entails designing products based on requirements and configuration rules^21,22^. Valid configurations result from adhering to defined combination rules^23^, necessitating systematic management of features and composition rules^15^. Feature Models (FMs) and Orthogonal Variability Models (OVMs) are key in variability management, with FMs representing functional commonalities and variabilities^24^. OVMs describe variant parts of base models^25^, with FMs being widely used in software product line (SPL) practices^26^ as part of the feature-oriented domain analysis (FODA) method.

**Figure 1:**
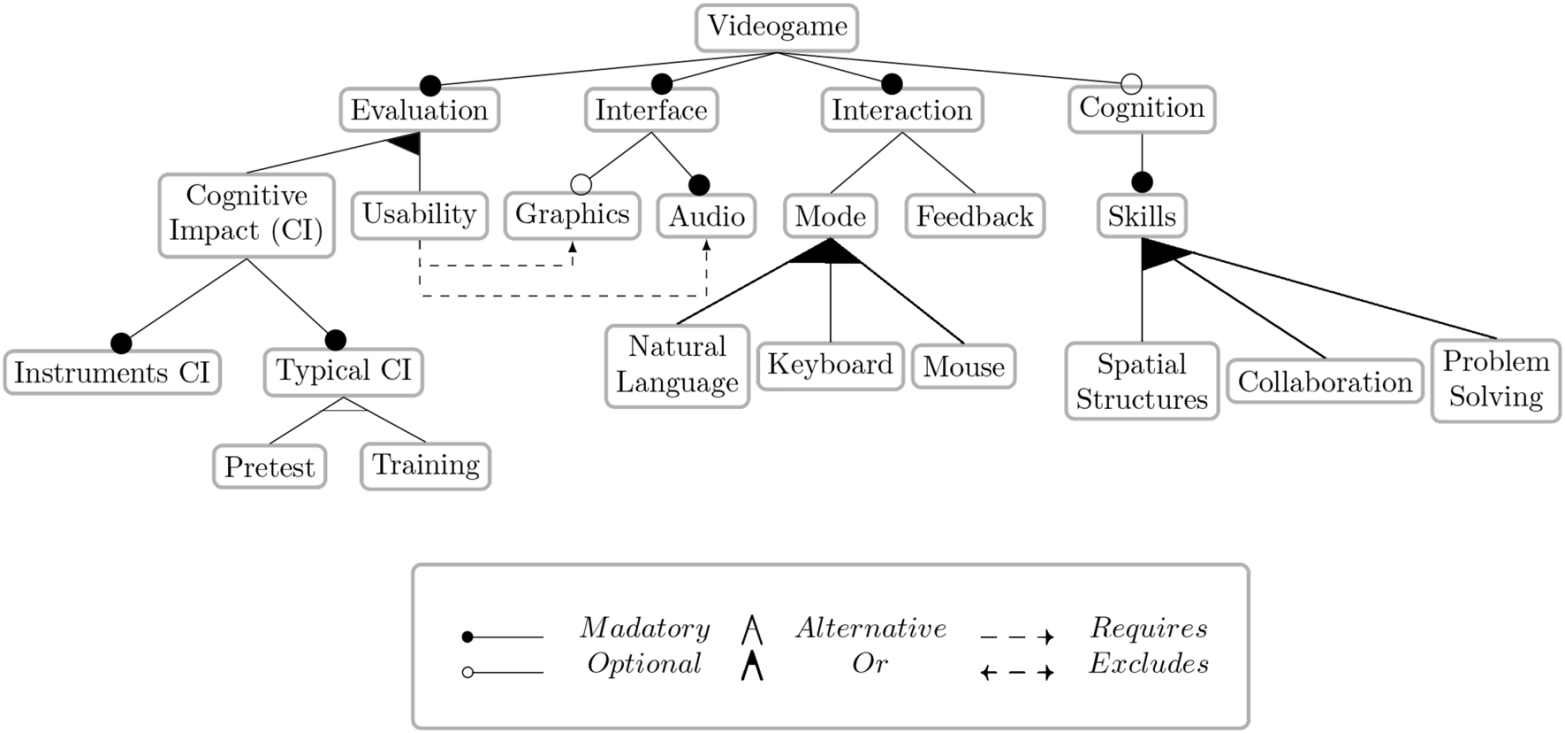
Feature model (FM) of a videogame products family.

A feature model (FM) defines a set of features and their relationships for defining valid feature combinations or products, that is, sets of features that respect the FM’s defined relationships. Figure 1 illustrates an FM for a videogame product family. We can appreciate different relationships between single-parent–child mandatory and optional features, set-parent–children alternative and optional (OR), and cross-tree requires and excludes constraints.

### Problem statement, goal and contributions

Product configuration enables mass customization production^23^. VIS instances in software engineering manage variability throughout development phases, crucial for meeting user expectations of adaptable software products^27^. Research on managing VIS variability exists, evident in works on Linux, Debian-based distributions, Android, and Drupal^28–32^, employing variability models for analysis.

Software product lines (SPLs) systematically manage commonalities and variabilities for software product configuration^33^. SPLs define domain engineering as analyzing and developing reusable functionalities and producing customized products based on user feature selection. Valid configuration definition in SPLs poses challenges due to increasing configuration knowledge base complexity^31^. Manual analysis of variability models, like feature models (FMs), is error-prone and time-consuming, particularly with growing model sizes^24^. For instance, Debian-based distribution models encompass around 28,000 variability points^30^. Automated analysis of feature models (AAFM) offers solutions to address these challenges^24^.

**Figure 2:**
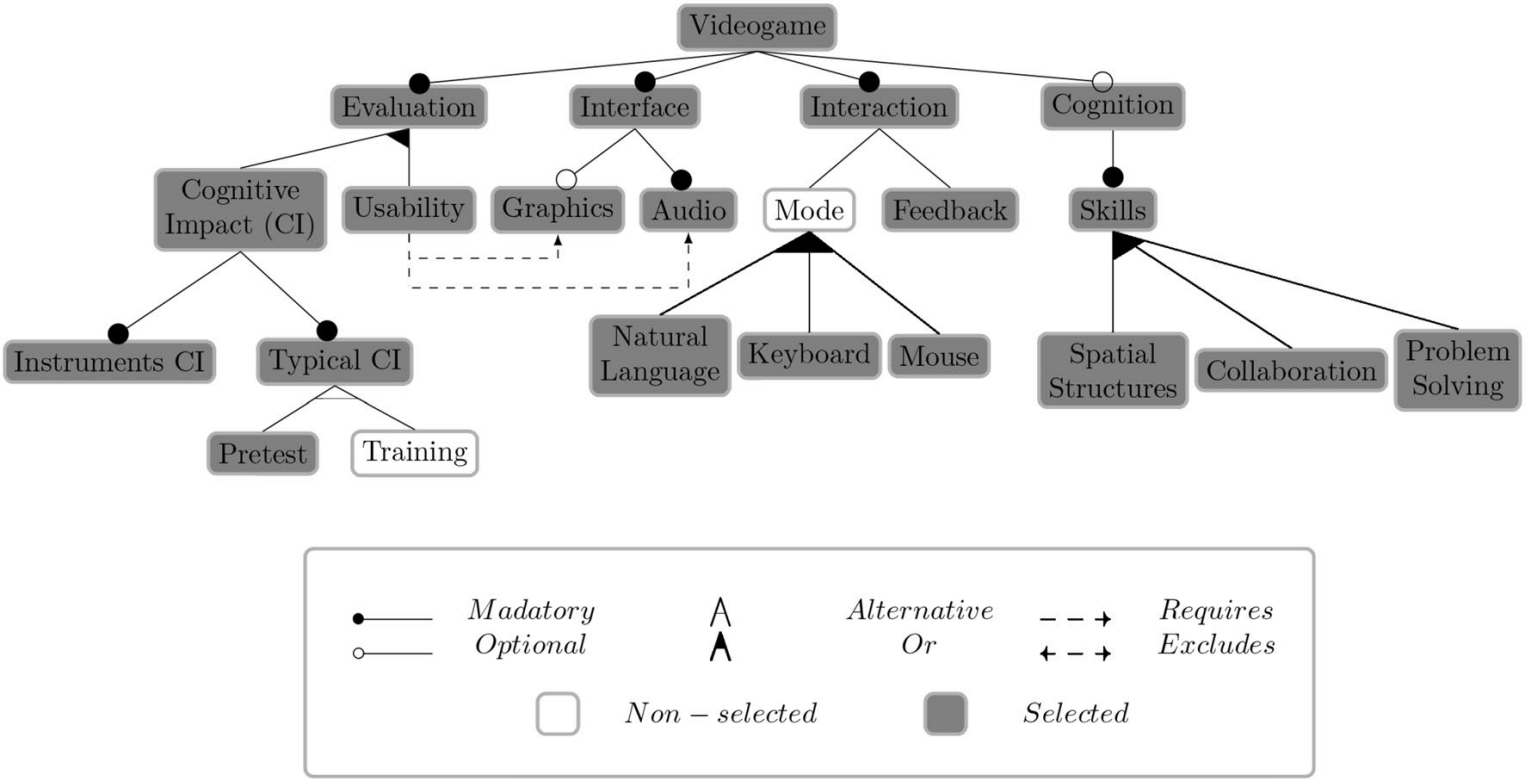
Feature model with a valid configuration example.

**Figure 3:**
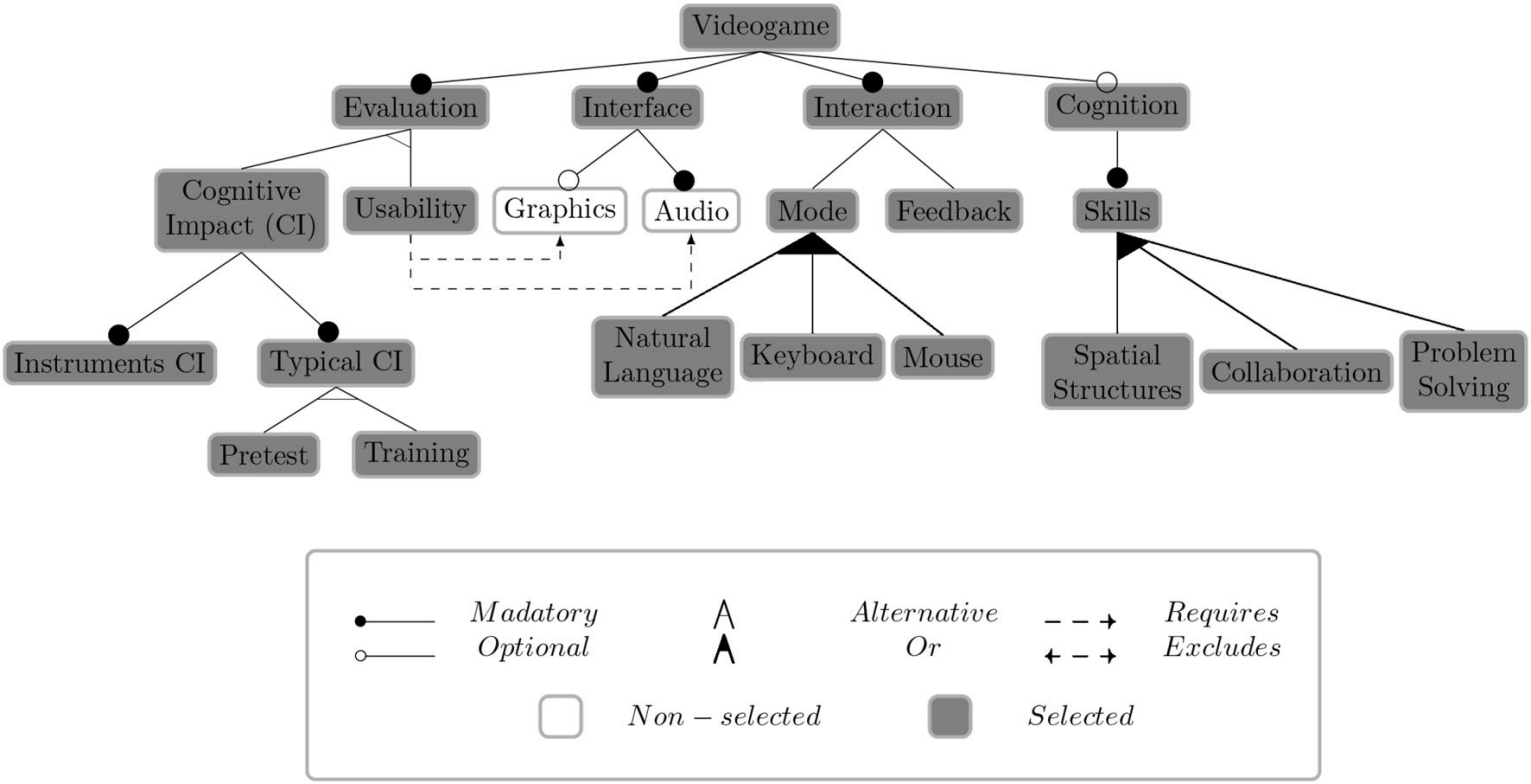
Feature model with a non-valid configuration example .

A configuration in a feature model (FM) represents a set of features, where each software variant within an FM corresponds to a valid configuration or product^34^. FMs provide a framework for organizing the configuration space and facilitating the construction of software variants by defining configuration options through interdependent features or functionalities. Thus, configuring FM products can be a valid or non-valid process; each selected option respects each configuration rule, or some rule is non-respected. By considering FM configuration rules, Figures 2 and 3 serve as illustrations of a valid and non-valid configuration in an FM, respectively.

Additionally, AAFM operations, crucial for resolving conflict-free FMs and configurations, represent high-value tasks. However, existing AAFM operations typically adhere to a sequential computing paradigm, limiting their scalability when confronted with large-scale and high-variability models, particularly in interactive application scenarios. While numerous algorithms and solutions for AAFM are documented in the literature, such as QuickXPlain^35^, FastDiag^36^ designed for detecting minimal conflicts and minimal-preferred diagnoses within conflicting sets of constraints, respectively, these recursive solutions are constrained by their inability to leverage additional resources for parallel and distributed computing, such as multiple cores or network technologies. In response to these limitations, AAFM solutions have been reimagined in their speculative versions, emphasizing their potential for enhanced computing efficiency in the product configuration of large-scale models.

The objective of this research is to assess and enhance parallel computing solutions in the context of product configuration in feature models (FMs). This encompasses both the evaluation of existing hardware and programming approaches for parallel computing solutions and the imperative need for optimizing current solutions to facilitate FM product configuration. This will be achieved through analyzing the functionality and computational performance of AAFM solutions utilized for minimal conflict detection and minimal diagnosis within product configuration, parallelized with QuickXPlain and FastDiag. Additionally, the aim is to highlight improvements in computational performance through the adaptation of speculative programming to commonly employed AAFM solutions. The goal is to minimize latency and optimize runtime, albeit at the expense of resource inefficiencies inherent in speculative programming.

Furthermore, for improving algorithms where traditional or direct parallelism is not viable, speculative programming enables pre-computation of potential execution paths, thus leveraging parallelism to achieve runtime enhancements.

To reach the previous goals, the rest of this paper is organized as follows. “Background”  describes and exemplifies the use of FMs. “Automated analysis of variability-intensive systems”  describes the Automated Analysis of Feature Model (AAFM) and the product configuration processes. That section also details the conflict detection and diagnosis operations with existing solutions and application results. “Speculative programming solutions”  describes the main differences and commonalities of the analyzed solutions for optimizing them and defining a general speculative solution. Hence, that section details the application results of ParallelQuickXPlain and ParallelFastDiag for the product configuration for a test set. “Threats to validity”  details a few practical issues of our research. The paper concludes by summarizing the benefits of our academic experience and detailing the motivation for continuing with it in the current and future years.

To improve the readability of the study, Table 1 presents the different acronyms in those topics with their meanings.

**Table 1:** Acronym list.

Acornym	Meaning
FM, FMs	Feature model, feature models
AAFM	Automated analysis of feature model
VIS	Variability intesive system
VM, VMs	Variability model, variability models
OVMs	Orthogonal variability models
SPL	Software product line
MCS	Minimal conflict set
SLR	Systematic literature review
MCS	Minimal conflict set
MD	Minimal diagnosis
FODA	Feature-oriented domain analysis

## Background

A feature model is an information model that represents the variant flexibility and maintainability for systems’ variability and configuration^22^. A feature is an abstraction of a prominent or distinctive user-visible aspect, requirement, quality, or functional characteristic of a family of software systems^37,38^; each feature constitutes a user-visible configuration option of the problem domain^39^. An FM is a tree-like structure commonly used to represent common and variable functionalities (features) and their relationships to the configuration of products in a software product line (SPL)^26^. Kang et al.^26^ introduced FMs in the FODA (Feature-Oriented Domain Analysis) method, and they are the “de facto” standard for describing common and variable features in system families^40,41^ regardless of their size because FMs facilitate the software reuse^42^.

An FM starts with the root feature. Each successively deeper level in the FM corresponds to a more fine-grained configuration option for product-line variants. Features are nodes of that tree, and their relationships are the edges (relationships and constraints) between features^37^. The relationships among features are of two types: structural relationships between a parent and its child features and cross-tree or cross-hierarchy constraints^37^. FMs represent an effective communication medium between customers and developers of SPLs^43^. As Benavides et al.^24^ describe, different FM dialects exist nowadays, such as basic FMs models, cardinality-based FMs, and extended FMs using feature attributes^44–46^.

### Basic feature models

An essential FM supports two types of relationships between features: structural relationships between parents and their child features and cross-tree constraints^24^. Thus, each non-root feature has a parent feature and is either part of a group or not. The following lines describe each type of FM relationship.

- Structural relationships between parents and their child features:
  - Mandatory: A mandatory relationship states that a parent feature requires its child. The top-left figure of Table 2 shows the graphic representation of a mandatory relationship between parent and child features.
  - Optional: An optional relationship states that a child feature may be or not be present (its parent feature does not require it). The top-right figure of Table 2 illustrates an optional relationship between parent and child features.
  - Set: A defined number of children’s features (sub-features) are selectable for products when their parent is selected. A cardinality relation [x, y] gives this number of features for x $<=$ y and y $<=$ number of child features in the set. Two cases are XOR (alternative) and Or (inclusive) sets.
    - Inclusive Or: At least one child’s features must be present. In this case, the cardinality relation is [1, n] (n corresponds to the number of child features). The middle-left figure of Table 2 illustrates an inclusive relationship between a parent feature and a set of children’s features. The middle-right row of Table 2 illustrates an alternative relationship between a parent feature and a set of children’s features.
    - Alternative XOR: Only one child feature must be present. The associated cardinality relation is [1, 1] in this case.
- Cross-tree constraints.
  - Requires: for two features, A and B, if A requires B, then A’s presence implies the presence of B in a product. The top division in the bottom-row of Table 2 illustrates a required cross-tree constraint relationship between a source feature A and a target feature B.
  - Excludes: for two features, A and B, if A excludes B, then A and B cannot be present in the same product. The bottom division of the bottom row of Table 2 illustrates an excludes cross-tree constraint relationship between features A and B.

**Table 2:** Feature model relations. .

Unary relations	Mandatory	
	Optional	
Set relations	Inclusive(OR)	
	Exclusive (XOR)	
Cross-tree constraints	Requires	
	Excludes	

More complex cross-tree relationships exist in the literature to define constraints in generic propositional formulas such as “A and not B implies C”^24^.

**Figure 4:**
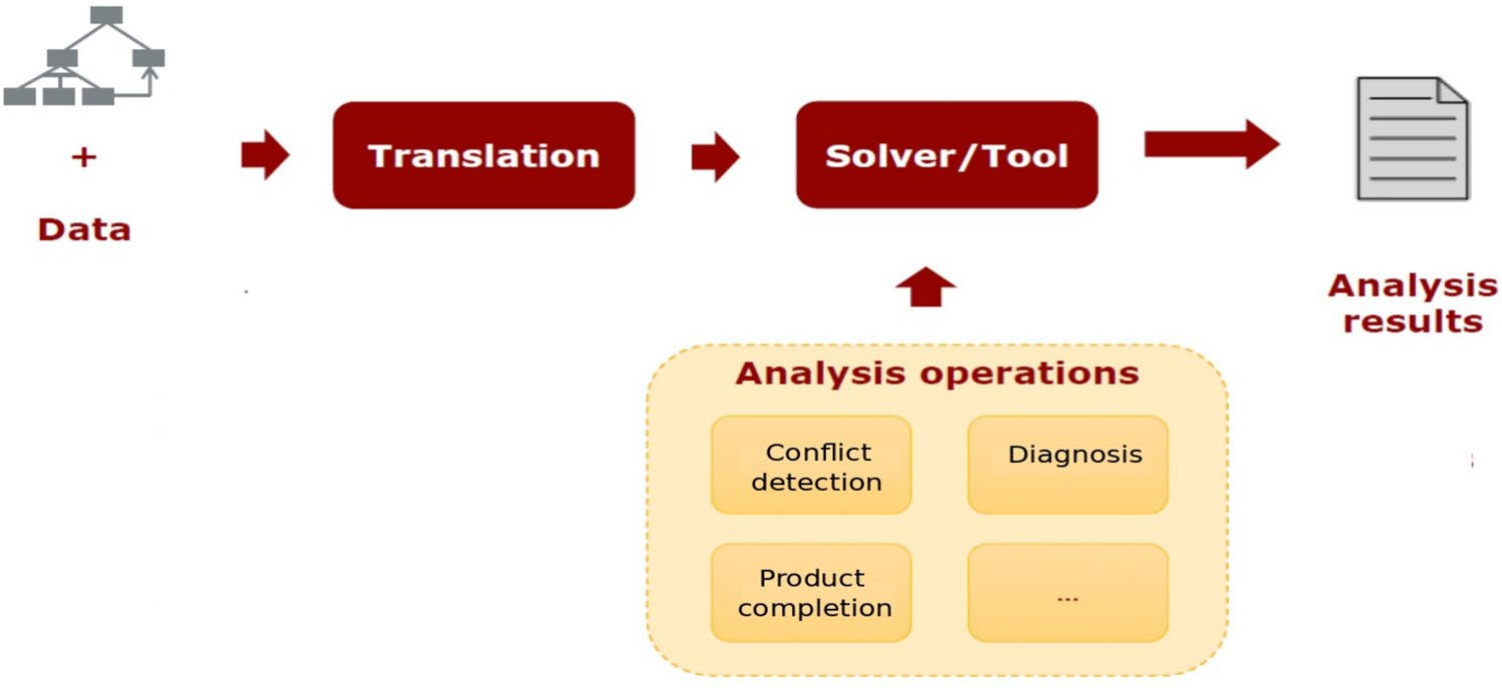
Automated analysis of feature models (AAFM) process^21^.

The application and analysis of FMs is a common approach to performing analysis tasks. Benavides et al.^24^ mention that the manual analysis of FMs is a time-demanding and error-prone activity, and the AAFM process permits solving those issues. The AAFM process starts by translating the FM and additional information, such as global restrictions, into logical constraints. Afterward, queries can proceed with the translated model using an off-the-shelf solver and other tools such as programming solutions, thus obtaining analysis results^47^. Figure 4 illustrates the AAFM process.

Such as Galindo et al.^47^ summarize six different variability facets that exist where the AAFM is currently applied: (i) product configuration and derivation, (ii) testing and evolution; (iii) reverse engineering; (iv) multi-model variability-analysis; (v) variability modeling, and; (vi) variability-intensive systems. The first AAFM application results in the most traditional usage of automated analysis mechanisms. This article aims to contribute to it.

Developing FM and product configurations without errors or conflicts requires identifying each conflict and the necessary steps to solve or diagnose it. Hence, conflict detection and diagnosis are operations needed to get conflict-free models. Completing an FM product configuration by hand also represents an error-prone and time-consuming task. Solutions for those tasks to work on large-scale models represent high-value tasks nowadays. AAFM solutions for product conflict detection, diagnosis, and completion already exist.

## Automated analysis of variability-intensive systems

The development process of a VIS considers identifying and representing the system’s components and relationships among those components as two core activities. The application and analysis of FMs is a common approach to performing those analysis tasks. Benavides et al.^24^ mention that the manual analysis of FMs is a time-demanding and error-prone activity, and the AAFM process permits solving those issues. The AAFM process starts by translating the FM and additional information, such as global restrictions, into logical constraints. Afterward, queries can proceed with the translated model using an off-the-shelf solver and other tools such as programming solutions, thus obtaining analysis results^47^.

For Galindo et al.^47^, six different variability facets exist where the AAFM is currently applied: (i) product configuration and derivation; (ii) testing and evolution; (iii) reverse engineering; (iv) multi-model variability-analysis; (v) variability modeling, and; (vi) variability-intensive systems. The first AAFM application results in the most traditional usage of automated analysis mechanisms. This article aims to contribute to it.

Developing FM and product configurations without errors or conflicts requires identifying each conflict and the necessary steps to solve or diagnose them. Hence, conflict detection and diagnosis are operations needed to get conflict-free models. Completing a product configuration of FM by hand also represents an error-prone and time-consuming task. Solutions for those tasks to work on large-scale models represent high-value tasks nowadays. AAFM solutions for product conflict detection, diagnosis, and completion already exist. The following sections describe an existing algorithm for detecting minimal conflict sets (MCS), a current algorithm for detecting minimal diagnosis (MD), and traditional approaches to complete product configurations.

### Product configuration solutions

Minimal conflict sets (MCS) detection: an MCS of a system represents a minimal set of constraints in conflict. For Definition 1^23^, it is necessary to identify the set of constraints *B* that represents a consistent background knowledge and the set of constraints *C* that is the suspected subject of a conflict search.

#### Definition 1

A set *AC* = *B* $\cup$ *C* = $\{c_{1}, c_{2}, ..., c_{n}\}$ represents the set of all constraints in the knowledge base; that is, *AC* is the union of the consistent knowledge base *B* and the suspicious set of constraints subject of conflict search *C*. Then, a conflict *CS* = $\{c_{a}, c_{b}, ..., c_{z}\}$ is a non-empty and non-consistent subset of *C*. *CS* is minimal if $\lnot \exists$ $CS'$ such that $CS' \subset CS$ *CS* is preferred if the order of its constraints follows a defined ranking of preferences.

QuickXPlain^35^ is an efficient approach to determining a minimal conflict set. QuickXPlain receives *C* as the set of suspicious constraints with conflict and *B* as consistent constraints of the background knowledge. Then, a conflict does not exist if *B* $\cup$ *C* is consistent or *C* is empty. On the other hand, QuickXPlain proceeds by returning the results of the function *QX*. *QX* receives the parameters *C* (initially the complete set of constraints with conflict), *B* (initially the knowledge base), and $B\delta$ (initially empty) that represents the last items added to *B*. Function *QX* follows a divide-and-conquer approach for conflict detection. Hence, $B\delta$ corresponds to the set of constraints added for reviewing the consistency of the knowledge base, and *C* is the set of constraints to continue analyzing if the current *B* is consistent. Algorithms 1 and  2 show the pseudo-code of the functions of QuickXPlain.

**Algorithm 1:**
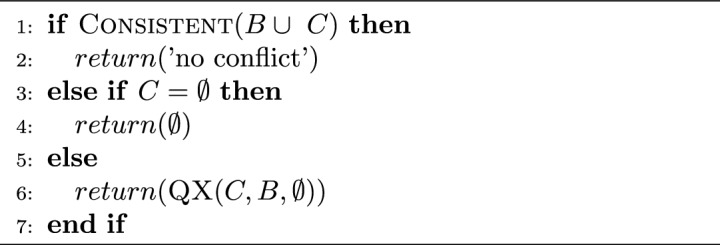
QuickXPlain(*C*, *B*) : *CS*.

**Algorithm 2:**
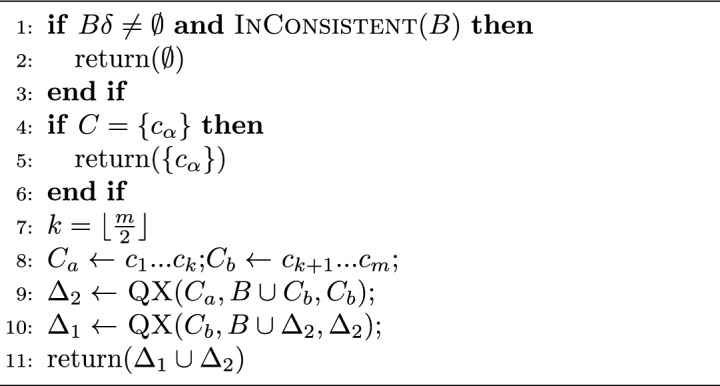
$$\textsc {QX}(C=\{c_1..c_m\},B,B\delta ): CS$$.

QuickXPlain permits determining one MCS per computation. Felfernig et al.^23^ indicate that we need to update adequately or delete one of the constraints of an MCS to solve it and, if the model is non-consistent yet, to apply QuickXPlain and repeat the process. When the resulting model is consistent, the updated constraints represent a diagnosis or solution for the model. A relevant step of function QX of QuickXPlain is step 1 to check for the inconsistency of set B, a task performed by an external tool like an SAT or CSP solver.

Assuming a splitting $k=\lfloor {\frac{m}{2}\rfloor }$ of $C=\{c_1..c_m\}$, the worst-case time complexity of QuickXPlain in terms of the number of consistency checks needed for calculating one minimal conflict is $2k \times log_2(\frac{m}{k}) + 2k$ where *k* is the minimal conflict set size and *m* represents the underlying number of constraints^35^. We should optimize the computing performance of consistency checks because they are the most time-consuming part of conflict detection.

Table 3 summarizes the results of the QuickXPlain performance analysis to identify a preferred minimal conflict of product configurations. Each entry represents the average runtime in *msec* for all knowledge bases with a preferred conflict set of cardinality *n* (1–16). We can appreciate that the time increases when more conflicts exist in the analyzed product configurations. For the mentioned issue that QuickXPlain identifies only one conflict that, after solving it, a new execution is necessary to determine the remaining one.

**Table 3:**
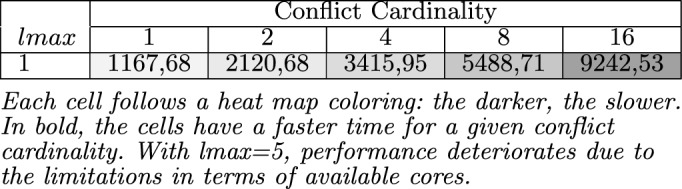
Avg. runtime (in ms) of QX when determining minimal conflicts.

Each cell follows a heat map coloring: the darker, the slower. In bold, the cells have a faster time for a given conflict cardinality. With lmax=5, performance deteriorates due to the limitations in terms of available cores.

### Minimal diagnosis detection

Identifying and solving conflicts one by one is necessary to obtain a conflict-free model: we need to identify a conflict first, adapt (update or eliminate) constraints of that conflict for its solution, and repeat this process until no more conflict exists, that is, until reaching a consistent model. The set of all the adapted constraints for getting a conflict-free model represents a diagnosis. Definition 2 formally defines the term diagnosis^23,48^.

#### Definition 2

A set *AC* = $\{c_{1}, c_{2},..., c_{n}\}$ represents the set of all constraints in the problem for diagnosis; that is, *AC* is the union of the consistent base knowledge *B* and the set of constraints subject of the conflict search *C*: *AC* = *B* $\cup$ *C*. Then, a diagnosis is a set of constraints $\Delta$ $\subseteq$ *C* such that (*B* $\cup$ *C* − $\Delta )$ results in a consistent or conflict-free set. $\Delta$ is minimal if $\lnot \exists$ $\Delta '$ such that $\Delta ' \subset \Delta$. A minimal diagnosis is of minimal cardinality if there does not exist a minimal diagnosis $\Delta '$ such as $|\Delta '|$ < $|\Delta |$.

A minimal diagnosis for the FM configuration of Fig. 3 has to consider solutions for each conflict. Hence, this example contains two diagnosis options. To get a conflict-free model, the user has to solve each diagnosis. Cases with multiple diagnosis instances exist, and determining all the diagnoses can be computationally expensive. Model constraints can be relevant for obtaining a preferred diagnosis. Obtaining all the diagnoses to look for the preferred one is a time-demanding and lost time activity since solving one diagnosis is enough for a conflict-free model. The next lines describe the FastDiag algorithm to determine a minimal preferred diagnosis.

FastDiag algorithm permits determining a preferred or leading diagnosis concerning a previously defined relevance order of constraints in the knowledge base. FastDiag follows the algorithmic structure and reasoning of QuickXPlain for a different purpose: diagnosis detection without calculating MCS instances. Hence, FastDiag is based on conflict-independent search strategies^49^. Algorithms 3 and  4 give the pseudo-code of FastDiag functions.

**Algorithm 3:**
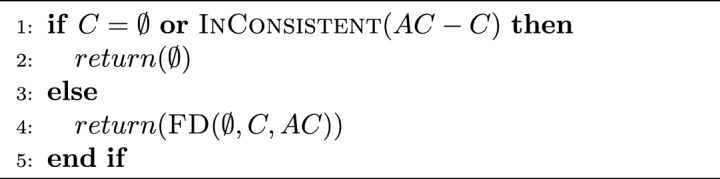
FastDiag(*C*, *AC*) :  *diagnosis* $\Delta$.

**Algorithm 4:**
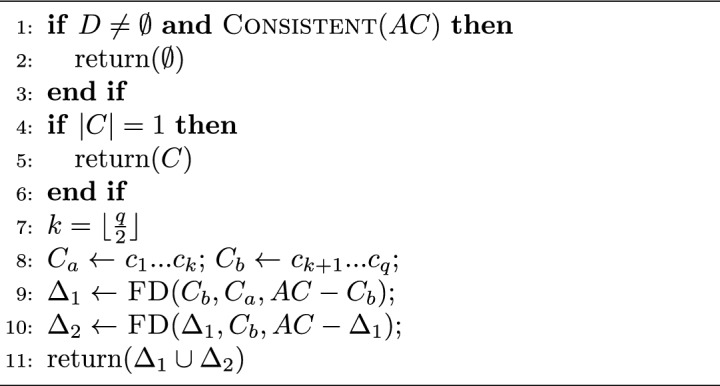
$$\textsc {FD}(D, C=\{c_1..c_q\},AC):$$ *diagnosis* $$\Delta $$.

Assuming that conflicts to diagnosis exist, If the conflict set *C* is non-empty, and *AC* without *C* is consistent, algorithm FastDiag calls and waits for the results of the recursive algorithm FD. FD first reviews the consistency of *AC* as a source of diagnosis. Because always *AC* contains *C* and does not contain *D*, *S* is the constraint set with conflicts, and *D* is empty; when *D* is not empty, and *AC* is consistent, *D* is the source of conflict. When that base case is not accomplished, either because *D* is empty (such as at the beginning) or *AC* is consistent (this is only possible after removing elements from $AC - D$ represents the last removed elements from *AC*), then *AC* is still in conflict, and *C* is a source of conflict. Then, FD reviews the size of *C* since if it were minimal (size 1), then *C* is the diagnosis. If *C* is not of minimal size, FD proceeds to partition *C* in the sets $C_1$ and $C_2$, of which the last one corresponds to the most preferred partition. Afterward, FD calls FD over $C_2$, $C_1$, and $AC - C_2$ to review if $C_2$ is the diagnosis source and, if not so, to continue reviewing $C_1$ with that goal.

Assuming a splitting $d=\lfloor {\frac{n}{2}\rfloor }$ of $S=\{s_1..s_n\}$, the worst-case time complexity of FD in terms of the number of consistency checks needed for calculating one minimal diagnosis is $2d \times log_2(\frac{n}{d}) + 2d$ where *d* is the minimal diagnosis set size and *n* represents the underlying number of constraints^49^. The runtime performance of the underlying algorithms must be optimized because consistency checks are the most time-consuming part of diagnosis detection.

Table 4 summarizes the results of the FastDiag performance analysis to identify a preferred minimal diagnosis of product configurations. Each entry represents the average runtime in *msec* for all knowledge bases with a preferred diagnosis set of cardinality *n* (1–16). We can appreciate a surprising time execution difference between the conflict and diagnosis detection; that is, algorithm FastDiag results more efficient than QuickXPlain even though they pursue different tasks. We can appreciate in Table 4 that the time increases when more conflicts exist in the product configurations because FastDiag requires identifying diagnosis of more cardinality.

**Table 4:**
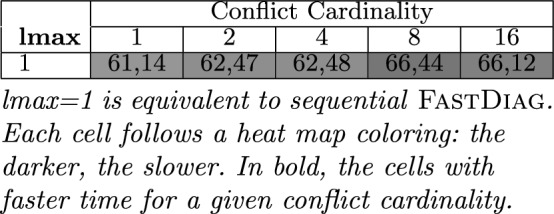
Avg. runtime (in ms) of FD (lmax = 1) for determining preferred diagnosis.

lmax = 1 is equivalent to sequential FastDiag. Each cell follows a heat map coloring: the darker, the slower. In bold, the cells with faster time for a given conflict cardinality.

In summary, existing product configuration solutions QuickXPlain and FastDiag are algorithms for identifying MCS and minimal diagnosis. Even though they are efficient sequential-computing solutions, such as Vidal et al.^15^ highlight, they are inadequate for large-scale FMs. The following section reviews the computing performance of those solutions. The QuickXPlain, FlexDiag, and data for experiments are available in https://github.com/cvidalmsu/A-Python-QX-implementation, and https://github.com/cvidalmsu/A-Python-FD-implementation, respectively.

## Speculative programming solutions

We can appreciate that QuickXPlain and FastDiag share a similar algorithm structure and behavior as the next lines describe.

- Both solutions start reviewing if a problem exists; that is, (i) if consistency exists in the base set plus the set of constraints to add to it, or (ii) if the base set minus the set of constraints to remove from it is inconsistent, to immediately return if some of them hold. The QuickXPlain algorithm appreciates as a second base case if the set of constraints to add to the base set is empty; that means, the base set is inconsistent, to return the empty set. The FastDiag algorithm considers that option in the first base case: if the set of constraints to remove from the base set is empty, then return the empty set.
- If none of the base cases is true in both solutions, they proceed with their main functions: (i) QuickXPlain receives the set of constraints to look for adding to the base set, the base set, and the set of constraints previously added to the base set (empty in the first call), whereas (ii) FastDiag receives the set of constraints already removed from the base set, the set of constraints to look for removing from the base set, and the base set.

The base set of QuickXPlain represents the primary structural model without additional constraints, like a feature model without options selection. On the other hand, the base set of FastDiag is the primary structural model, plus additional constraints. Hence, the primary function of QuickXPlain looks to find inconsistencies by adding elements to the base set. In contrast, the primary function of FastDiag looks to find consistency by removing elements from the base set. In summary, QuickXPlain and FastDiag work on a set of base constraints, a set of constraints to add to or quit from the base set, and the last set added to or quit from that set, respectively.

The following lines describe the main elements we considered to design and implement speculative parallel versions of QuickXPlain and FastDiag solutions.

### Looking for QuickXPlain and FastDiag optimizations

#### Consisteny checking

The consistency checking task is a high-cost, recurrent, and sometimes repetitive operation in QuickXPlain and FastDiag. Then, we look to avoid that operation repetition by storing the consistency checking in a hash table to check for its existence first and get that value, or, otherwise, to apply the consistency checking operation and store it (memorization process). This improvement could also be used to improve the computing performance of the QuickXPlain and FastDiag solutions. Algorithms 5 and 6 illustrate the inconsistency and consistency checking for QuickXPlain and FastDiag, respectively.

**Algorithm 5:**
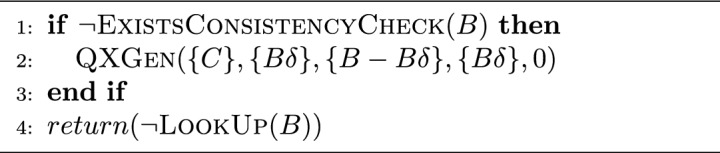
Inconsistent($$C,B,B\delta $$):*Boolean*.

**Algorithm 6:**
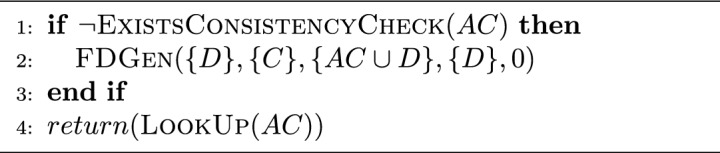
Consistent(*D*, *C*, *AC*):*Boolean*.

#### Speculative paths

Algorithms 2 and  4 follow a divide-and-conquer approach and behave similarly concerning their specific goals: minimal conflict and diagnosis detection, respectively. In addition to avoiding the recalculation of consistency or inconsistency checking previously checked, a general speculative approach should perform that checking process asynchronously. Then, both solutions must consider that case before speculating regarding the true or false checking value because QuickXPlain and FastDiag, after the respective consistency and inconsistency checking, the analyzed set minimality is reviewed. Algorithm 7 details the general steps of ParallelQuickXPlain^50^ and ParallelFastDiag^15,51^ solutions (Gen for QXGen and FDGen).

A relevant element to consider is the speculation level reached for parallelism capacity. Hence, before evaluating the previously described steps, a first base case is needed to check that situation. |*f*(*X*)| denotes the number of constraints $c_i$ in *X*. The AddCC function triggers an asynchronous task that is in charge of adding consistency checks (parameter of AddCC) to a LookUp table and issuing the corresponding solver calls (memorization process). *lmax* is a global parameter that defines the maximum search depth of one activation. Each Gen recursive call is executed in parallel (a new parallel task is created to execute that function). Thus, by each Gen execution, two new Gen tasks could be executed. Hence, parallel hardware capacity is crucial in speculating eventual execution flows.

Concerning the consistency checking step of original QuickXPlain and FastDiag, our solution proposal does not parallelize the nature of that task; we applied speculative computing for the execution of each consistency checking step of QuickXPlain and FastDiag, not in the steps of the consistency checking process itself.

**Algorithm 7:**
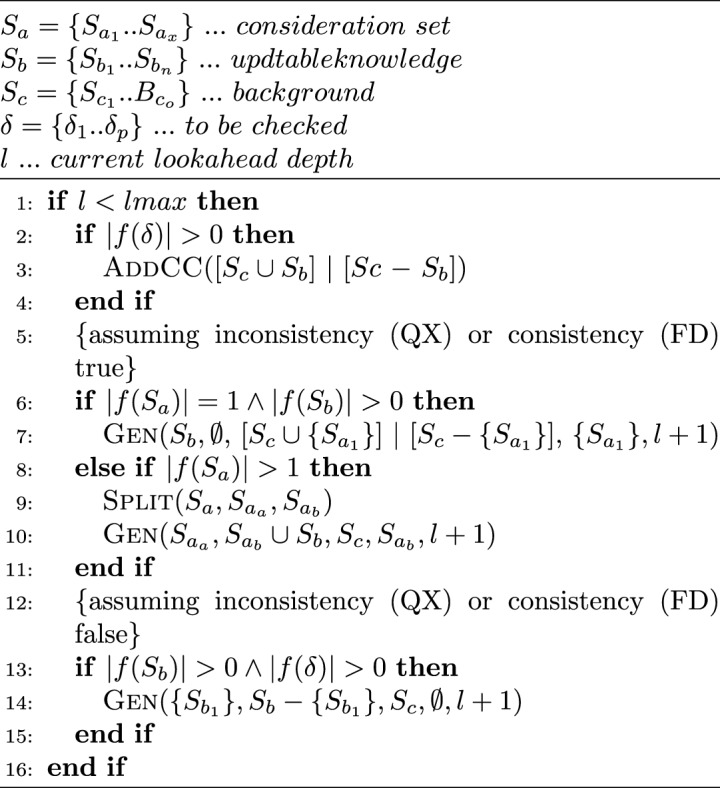
Gen($$S_a$$, $$S_b$$, $$S_c$$, $$\delta $$, *l*).

The following subsections describe the specialization of Algorithm 7 for ParallelQuickXPlain and ParallelFastDiag and application results on a case study.

### Parallel QuickXplain

Because the consistency verification represents a high-cost computing task, our speculative computing approach looks to parallelize the consistency checks in QX by substituting the *direct* solver call Inconsistent(B) in QX with the activation of a lookahead function (QXGen) in which consistency checks are not only triggered to provide feedback to QX requests directly. Moreover, our speculative approach provides fast answers for consistently checking potentially relevant in upcoming states of a QX instance. We follow the principles of speculative programming^52^: we start calculating consistency checks that could be useful in the future to anticipate resource-intensive reasoning tasks for reaching more efficient computing results. The drawback is that we use some computation resources that will be wasted if the pre-calculation is finally not used. Therefore, the challenge in this kind of technique is to find algorithms that can anticipate as many reusable calculations as possible while reducing calculation tasks that are not reusable.

As Vidal et al.^50^ describes, the QXGen function is based on the idea of issuing recursive calls and adapting the parameters of the calls depending on the two possible situations (1) *consistent*($B\delta \cup B$) and (2) *inconsistent*($B\delta \cup B$).

The experimentation was conducted based on a *Python3* implementation of the QuickXPlain algorithm and the parallelized QuickXPlain (QX) version presented in this research. For the implementation, we used the *multiprocessing* *Python* package for running parallel tasks. For representing our test knowledge bases and conducting the corresponding consistency checks, we used Sat4J^53^ as it is one of the most used solvers integrated in many software (product line) engineering tools such as FeatureIDE^54^, FAMA^55^, FAMILIAR^56^ among others^57–59^. Python was used for its parallelization capabilities while Sat4J was one of the most used solvers in the SPL community. Nevertheless, any other technologies could have been used.

**Table 5:**
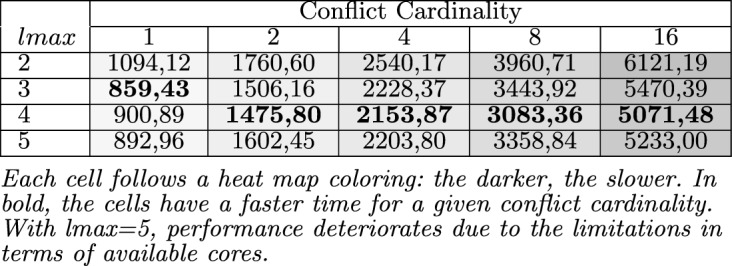
Avg. runtime (in ms) of parallelized QX when determining minimal conflicts.

Each cell follows a heat map coloring: the darker, the slower. In bold, the cells have a faster time for a given conflict cardinality. With lmax=5, performance deteriorates due to the limitations in terms of available cores.

Table 5 summarizes the results of our QXGen performance analysis. Compared to Table 3, on average, the runtime needed by standard QuickXPlain ($lmax=1$) to identify a preferred minimal conflict of cardinality 16 is $1.82\times$ higher compared to a parallelized solution based on *QXGen* ($lmax=4$). In Table 5, each entry represents the average runtime in *msec* for all knowledge bases with a preferred conflict set of cardinality *n*, where the same set of knowledge bases has been evaluated for *lmax* sizes 2–5. Although speculative computing and memorization allow for improving the execution speed of conflict detection solutions, that is not a computing efficiency at all for the required memorization that can be critical for larger models and deeper speculation levels. Nonetheless, solution speed is more relevant for interactive scenarios, and ParallelQuickXPlain allows improving the QuickXPlain ones.

**Figure 5:**
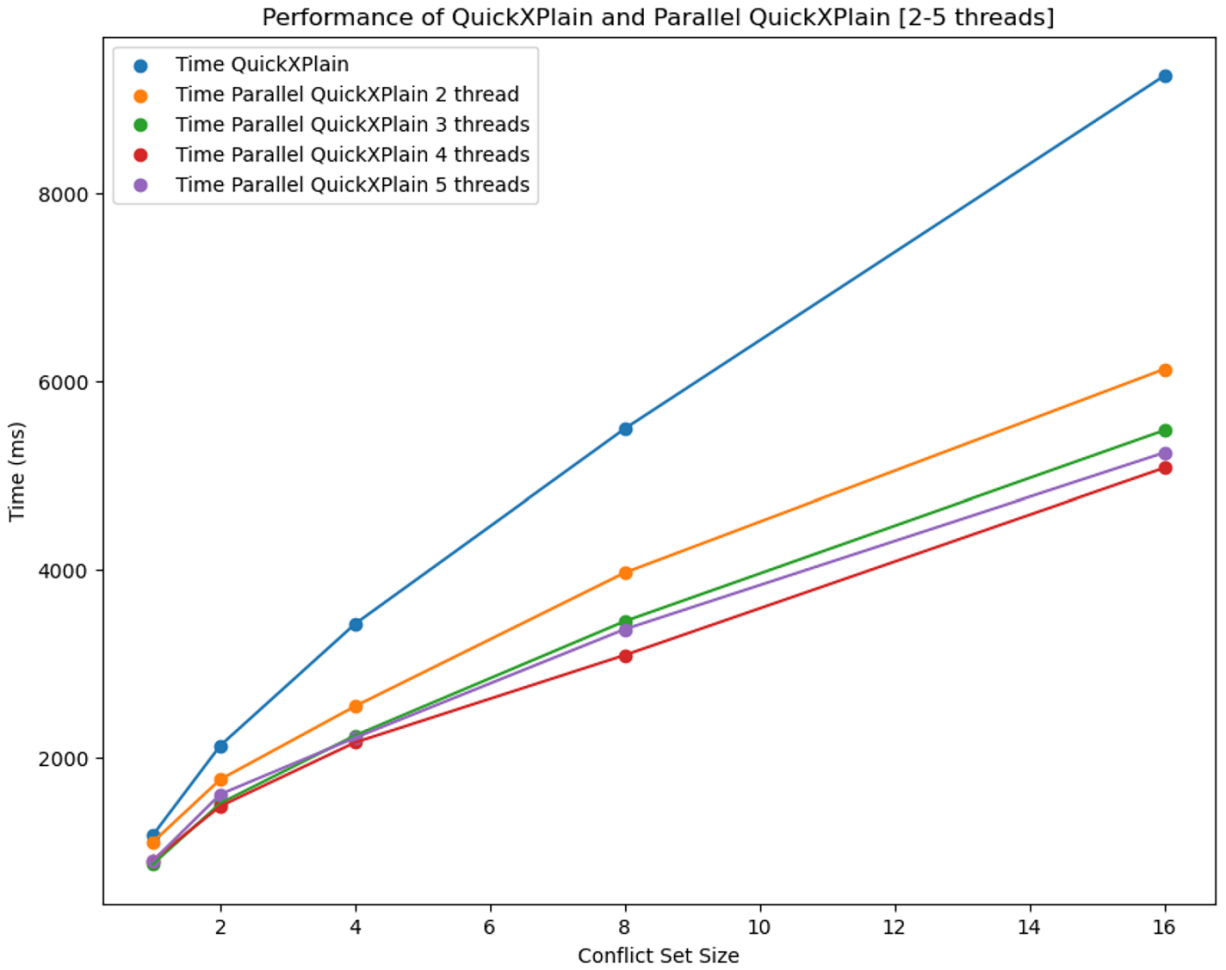
Performance of QuickXPlain vs ParallelQuickXPlain with 2 to 5 threads.

It can be observed that with an increasing *lmax*, the performance of QX increases. However, with $lmax=5$, a performance deterioration can be observed, which can be explained by the number of pre-generated consistency checks starting to exceed the number of physically available processors. In the line of our algorithm analysis, the number of relevant consistency checks that can be performed with $lmax=5$ is between 5 and 3. Considering the overheads for managing the parallelized consistency checks, the results support our theoretical analysis of QXGen. Figure 5 depicts the same results.

### Parallel FastDiag

Such as Felfernig et al.^23^ argue that consistency checking CC is an expensive computing step. Similar to the Parallel QuicXplain solution, our speculative computing approach to parallelizing the CC in FD substitutes the *direct* solver call consistent(AC) with the activation of a lookahead function (FDGen) in which consistency checks are not only triggered to provide feedback to FD requests directly, but also to be able to provide fast answers for consistency checks potentially relevant in upcoming states of a FD instance. We again follow the speculative programming principles^52^: we start calculating consistency checks that could be useful in the future. The advantage is that we can anticipate resource-intensive reasoning tasks. The drawback is that we use some computation resources that will be wasted if some pre-calculation is finally not used. Therefore, the challenge in this kind of technique is finding algorithms that can anticipate as many reusable calculations as possible while reducing the calculation tasks that are not reusable.

As Vidal et al.^15^ and Le et al.^51^ remark, in the proposed parallelized variant of ParallelFastDiag, CC is activated by FD with Consistent(*D*, *S*, *AC*). This also activates FDGen that starts to generate and trigger (in a parallelized fashion) further CC instances that might be relevant in upcoming FD phases. For describing FDGen, we employ a two-level *ordered set* notation which requires to embed the FD *D* into $\{D\}$, *S* into $\{S\}$, and *AC* into $\{AC\}$. In FDGen, *D*, *S*, and *AC* are interpreted as *ordered sets*.

We experimented based on the implementation in *Python3* of FastDiag and ParallelFastDiag. We used the *multiprocessing* *Python* package for running parallel tasks. We used Sat4J^53^ for representing our test knowledge bases and conducting the corresponding consistency checks since it is one of the most used solvers integrated in many software (product line) engineering tools such as FeatureIDE^54^, FAMA framework^55^, FAMILIAR^56^ among others^57–59^. Nonetheless, we could use any other technology for writing and reasoning on AAFM solutions.

**Table 6:**
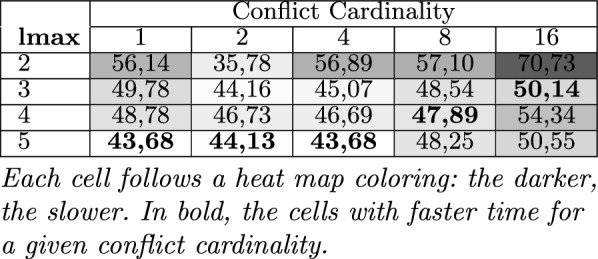
Avg. runtime (in *msec*) of FD (lmax=1) and parallelized FD ($lmax>1$) for determining preferred diagnosis..

Each cell follows a heat map coloring: the darker, the slower. In bold, the cells with faster time for a given conflict cardinality.

Table 6 summarizes the performance and analysis results of FastDiag and FDGen. On average, the runtime needed by standard FastDiag ($lmax=1$ in Table 4) to identify a preferred minimal diagnosis for conflict of cardinality 16 is $23,54\%$ slower compared to a parallelized solution for the same purpose based on FDGen ($lmax=5$). In Table 6, each entry represents the average runtime in *msec* for all knowledge bases with a conflict set of cardinality *n*, where the same set of knowledge bases has been evaluated for *lmax* sizes 2–5. As in the ParallelQuickXPlain and QuickXPlain results comparison, although speculative computing and memorization allow for improving the execution speed of conflict detection solutions, that is not a computing efficiency at all for the required memorization that can be critical for larger models and deeper speculation levels. Nonetheless, solution speed is more relevant for interactive scenarios, and ParallelFastDiag allows improving the FastDiag ones.

**Figure 6:**
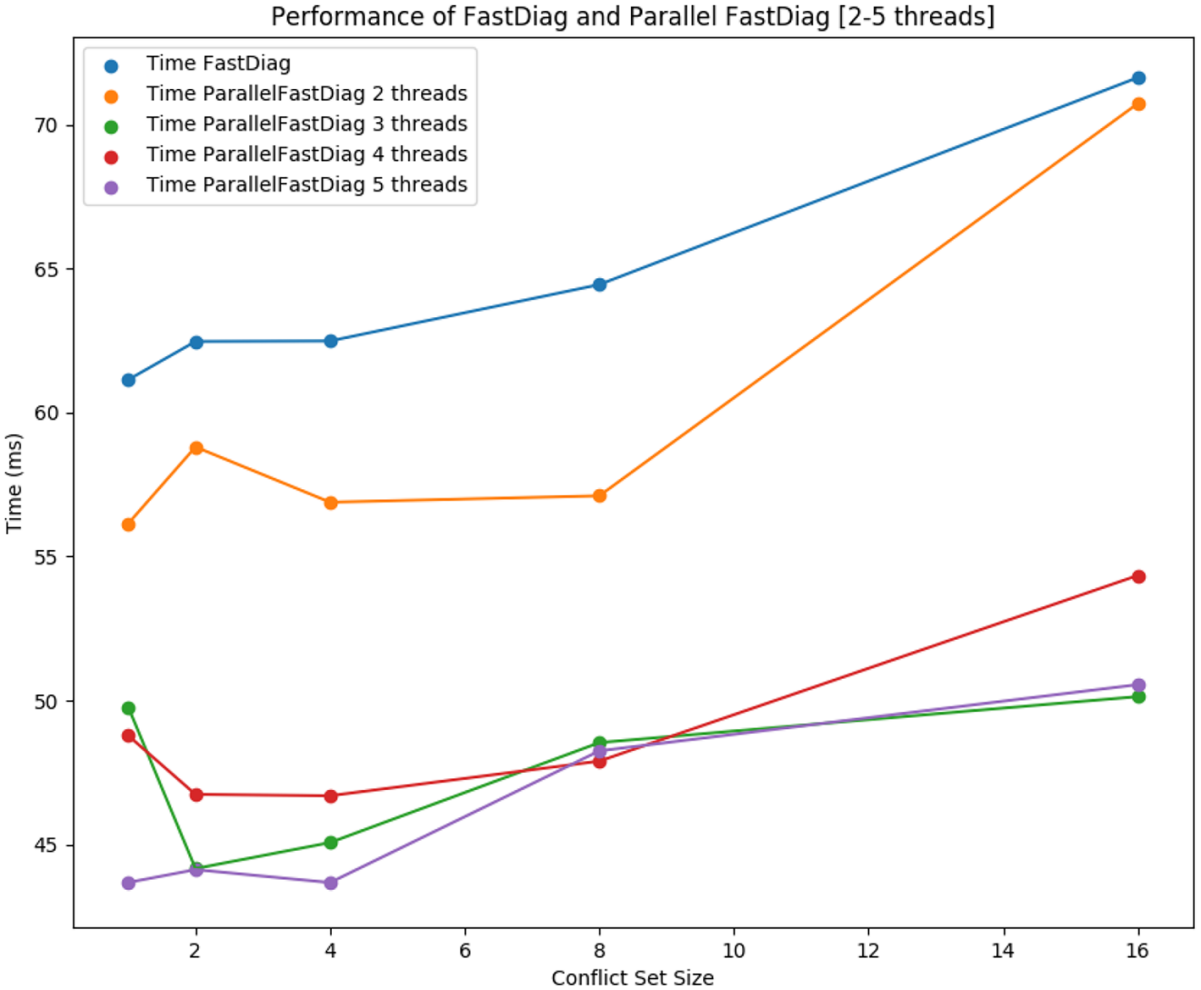
Performance of FastDiag vs ParallelFastDiag with 2 to 5 threads.

We can observe that with an increasing *lmax*, the performance improvement of FD increases with a few exceptions: the solution for four threads is the best for models with eight conflicts, and the solution for three threads is the best for models with sixteen conflicts. A deterioration can exist with $lmax=4$ and $lmax=5$ because the number of pre-generated consistency checks starts to exceed the number of physically available processors. The obtained results support our theoretical analysis of FDGen, taking into account the overheads for managing the consistency checks in parallel. Figure 6 illustrates the performance results of Table 6. The performance improvement of ParallelFastDiag presents a scalability tendency even though it is not as notorious as for ParallelQuickXPlain. After reviewing the results, some conflicts are solvable by updating only one or a few constraints. Then, finding a conflict set with various conflicts can require more computation.

## Discussion

### Execution environment

All experiments reported were conducted using an AMD EPYC 7571 machine with a CPU with eight cores and 2.60 GHz. Each core maintained up to two threads, which means that 16 cores could be simulated using hyper-threading. It had 64 GB of RAM.

For evaluation purposes, the experiments of this study did not involve human participants or related data. We generated configuration knowledge bases (feature models) from the publicly available Betty tool suite^45^, which allows for systematic testing of different consistency checking and conflict detection approaches for knowledge bases. The knowledge base instances that were selected for our evaluation had around 1.000 binary variables (derived from the 1.000 features used) and also varied in terms of the number of included constraints depending on the different feature relationships and the total of derived clauses (around 1600 SAT clauses in the generated CNF files). Based on these knowledge bases, we randomly generated requirements ($c_i \in C$) that covered $10\%$ of the variables included in the knowledge base. These requirements have been generated to analyze conflict sets of different cardinalities. We also shuffled the background set to get different orders because this can affect the number of consistency checks needed. In this research work, all methods and experiments were carried out as part of the research team, taking into account their previous research work and studies. No regulation was required since we did not involve humans and their data.

This article highlights the usability of speculative programming for optimizing the diagnosis and other conflict detection operations such as MergeXplain^60^. Vidal et al.^61^ showed the usability and efficiency of applying diagnosis solutions such as FastDiag for product completion.

One issue of speculative programming is the generation of non-effective speculations, that is, computations with non-usable results. Thus, applying speculative programming demands computing effective speculations as much as possible, speculations with a high grade of effectiveness. That requires a deep study of the current solutions to define and compute speculation strategies that can guarantee their effectiveness. To show the functionality and evaluate the performance of our solutions, we implemented them using Python and FAMA^33^.

## Threats to validity

This work presents the application of speculative programming to get better computing results with relevant operations for the Automated Analysis of Product Configuration of Feature Models in parallel regarding their sequential version. We can appreciate parallelism in solutions with dependent steps. Nonetheless, it is necessary to discuss the following practical issues:

- We implemented our solutions to run in Python and FAMA^33^. For executing QuickXPlain and FastDiag, Python and FAMA should be in the computer. That seems not to be a problem because Python in 2023 is one of the most used programming environments, and FAMA is freely accessible online.
- We worked with generated FMs by the use of Betty. Product configuration solutions can be more precise in inaccurate models and configuration cases. Nonetheless, the generated models are adequate for the simulation goal.
- The effects of speculative computing strongly depend on the hardware; that is, on the number of cores and, in our solution, probably also on the available memory due to the need to fit several SAT solvers. Thus, computing results of our solutions ParallelQuickXPlain and ParallelFastDiag, and of their base solutions QuickXPlain and FastDiag depends on the execution hardware configuration.
- We defined adequate computing solutions for conflict detection and diagnosis in the product configuration of large-scale feature models, ParallelQuickXPlain and ParallelFastDiag, respectively.

## Conclusion

This article describes the speculative programming application to parallelize two classical and sequentially efficient AAFM solutions to enable them for the automated analysis of large-scale feature models. The obtained results demonstrate the parallelism computing scalable improvements. This article reviewed the functionality, computing performance, and main details of QuickXPlain and ParallelQuickXPlain for conflict detection and FastDiag and ParallelFastDiag for diagnosing the product configuration of small-scale and large-scale products.

We provided the base and highlighted the speculative programming approach as an algorithmic optimization technique applicable for optimizing sequential solutions to work on the product configuration of large-scale products. With more detail,

1. We recognized that conflict detection is a base step for solving configuration issues. We found that QuickXPlain represents an efficient solution for detecting minimal preferred conflict. Although QuickXPlain uses an efficient divide-and-conquer algorithmic approach, analyzing large-scale FM and configurations takes a long time. Moreover, for its sequential nature, QuickXPlain cannot use computing resources, such as multiple cores for parallel computing. This article parallelized QuickXPlain to develop a more efficient solution for detecting conflicts in large-scale configuration scenarios. Our analysis found a costly operation step that uses data from the previous executions in the QuickXPlain functioning. We pre-calculate that operation by applying speculative computation to look for improvements. Thus, ParallelQuickXPlain was born. The obtained results validated the improvements of ParallelQuickXPlain regarding traditional QuickXPlain for analyzing large-scale FM and configurations.
2. We found that FastDiag represents an efficient solution for detecting minimal preferred diagnosis using an efficient divide-and-conquer algorithmic approach. However, FastDiag takes a long time to analyze large-scale FM and configurations, and it cannot use computing resources, such as multiple cores for parallel computing, for its sequential nature. Hence, we parallelized FastDiag for getting a solution for diagnosis in large-scale configuration scenarios as our second research goal. Like in the analysis of QuickXPlain, our analysis found a costly operation step that uses data from the previous executions in the FastDiag functioning. We pre-calculate that operation by applying speculative computation to look for improvements. Thus, ParallelFastDiag was born. The obtained results validated the efficiency of ParallelFastDiag regarding traditional FastDiag for analyzing large-scale FM and configurations.

## Data Availability

https://github.com/cvidalmsu/A-Python-QX-implementation and https://github.com/cvidalmsu/A-Python-FD-implementation.

## Code availability

https://github.com/cvidalmsu/A-Python-QX-implementation and https://github.com/cvidalmsu/A-Python-FD-implementation.
